# Recombination facilitates neofunctionalization of duplicate genes via originalization

**DOI:** 10.1186/1471-2156-11-46

**Published:** 2010-06-09

**Authors:** Cheng Xue, Ren Huang, Shu-Qun Liu, Yun-Xin Fu

**Affiliations:** 1 GuangDong Institute for Monitoring Laboratory Animals, Guangzhou, China; 2 Key Laboratory of Laboratory Animals in GuangDong, Guangzhou, China; 3 Laboratory for Conservation and Utilization of Bio-resources, Yunnan University, Yunnan, China; 4 Human Genetics Center, School of Public Health, University of Texas at Houston, Houston, Texas, USA

## Abstract

**Background:**

Recently originalization was proposed to be an effective way of duplicate-gene preservation, in which recombination provokes the high frequency of original (or wild-type) allele on both duplicated loci. Because the high frequency of wild-type allele might drive the arising and accumulating of advantageous mutation, it is hypothesized that recombination might enlarge the probability of neofunctionalization (P_neo_) of duplicate genes. In this article this hypothesis has been tested theoretically.

**Results:**

Results show that through originalization recombination might not only shorten mean time to neofunctionalizaiton, but also enlarge P_neo_.

**Conclusions:**

Therefore, recombination might facilitate neofunctionalization via originalization. Several extensive applications of these results on genomic evolution have been discussed: 1. Time to nonfunctionalization can be much longer than a few million generations expected before; 2. Homogenization on duplicated loci results from not only gene conversion, but also originalization; 3. Although the rate of advantageous mutation is much small compared with that of degenerative mutation, P_neo _cannot be expected to be small.

## Background

Gene duplication is the most common way of evolving new genes [1-4], but it is still argued how new genes evolve from duplicate genes in detail [5-7]. Ohno (1970) proposed that new genes might be fixed at one of duplicated loci by genetic drift, which was called neofunctionalization. Because degenerative mutations might also be fixed on the duplicated loci (called nonfunctionalization) and the occurring rate of degenerative mutation is usually much larger than that of advantageous mutation, the evolutionary fate of most duplicate genes is nonfunctionalization [8]. However, it has been observed that many duplicate genes are retained in some genomes, such as in tetraploid fish [9], *Xenopus Laevis *[10], and yeast *Saccharomyces cerevisiae *[4,11,12]. So it is necessary to explain these observations reasonably.

Assuming double null recessive selection and unlinked duplicated loci, Walsh (1995 and 2003) modeled the state of the population as a three-state (wild-type, degenerative and advantageous alleles) Markov chain, and thus calculated the probability (P_neo_) that the advantageous allele will fix before the nonfunctional allele does [13,14]. Under weak positive selection (roughly Ns < < 1), P_neo _was given by(1)

where EXP is the exponential function, ρ is the ratio of advantageous mutation rate (μ_neo_) to degenerative mutation rate (μ_non_), N is effective population size, and s is positive selection coefficient. Under strong positive selection, this formula is corrected,(2)

And Walsh (2003) also suggested that recombination might enlarge P_neo_, but he neither provided theoretical evidences, nor gave further explanation or hypothesis [14]. Recently Xue and Fu observed a mathematical process that we named originalization during the evolution of gene duplication under recombination, which can explain this suggestion [15]. During originalization, under purifying selection recombination results in the higher frequency of the original allele on both duplicated loci, so mean time to nonfunctionalization (T_non_) is prolonged. And it was hypothesized that prolonged T_non _and high frequencies of the wild-type allele might confer the arising and accumulating of advantageous alleles in the population, so that P_neo _might become larger [15-17].

In this article, we will test the hypothesis of enlarged P_neo _for unlinked gene duplication by originalization, and explore the underlying mechanism. Our results show that under stronger positive selection (Roughly Ns > 0.5) and in larger populations (Roughly N μ_non _> 0.1) recombination not only enlarges P_neo_, but also shortens mean time to neofunctionalization of duplicate genes (T_neo_). Therefore, through originalization recombination facilitates neofunctionalization of duplicate genes.

## Results

### Assumptions and notations

Assume that the duplicate genes originated from polyploidization, such as ancient whole genomic duplication (WGD), so that the effects of some genetic forces on small segmental duplications, such as unequal crossing over and gene conversion, are ignored, as assumed in previous theoretical studies on neofunctionalization of duplicate genes [13,14].

Assume in a random mating, diploid population, chromosomal haplotype is used to represent various genotypes of individuals [15,16]. Considering advantageous and degenerative mutations, there are three types of alleles at one of duplicated loci: wild-type allele (denoted as a character '0'), degenerative allele (denoted as a character '1'), and advantageous allele (denoted as a character '2'). In this way, there are nine possible types of chromosomal haplotypes in the population, namely, "00", "01", "02", "10", "11", "12", "20", "21" and "22", respectively.

We use the DNR (double null recessive or haplosufficient) and haploinsufficient (HI) selective models presented in our previous studies [15,16]. Under the DNR selective model, individuals with no wild-type allele at both of duplicated loci are invalid (relative fitness is 0), for example, individuals with chromosomal haplotypes "11" and "11", or "12" and "22". Under the HI selective model individuals with at least two copy of wild-type alleles on duplicated loci are valid. Assume mutation rates are the same on the duplicated loci; Transition (or mutation) from original allele to degenerative or advantageous allele is irreversible; Mutations from degenerative to advantageous and from advantageous to degenerative are ignored.

Under these assumptions, we report mean time to neofunctionalization (T_neo_) under the model only involving neofunctionalization and P_neo _under the model involving neofunctionalization and nonfunctionalization (details of the models are shown below).

### Mean time to neofunctionalization for gene duplication

#### Model

Let's consider a very simple model only involving neofunctionalization for duplicate genes at first. In this model, there are only four types of possible chromosomal haplotypes in the population, "00", "02", "20" and "22", and their frequencies in the population are denoted as x_0_, x_1_, x_2 _and x_3 _respectively. Because x_0_+x_1_+x_2_+x_3 _= 1, three of these four frequencies are independent and x_0_, x_1_, x_2 _are focused. Assume advantageous mutations are additive with fitness 1+ks for k advantageous allele(s) totally at duplicated loci. Fitnesses of individuals with various genotypes are shown in Table 1. Thus, without considering genetic drift (i.e. in an infinite population), differential changes of chromosomal haplotype frequencies at every generation, are given by a group of ordinary differential equations (ODEs),(3)

**Table 1:** Fitnesses of individual genotypes for neofunctionalizaion of gene duplication *

Chromosomal Haplotypes	"00"	"02"	"20"	"22"
"00"	1	1+s	1+s	1+2s
"02"	1+s	1+2s	1+2s	(1-s_1_)(1+3s)
"20"	1+s	1+2s	1+2s	(1-s_1_)(1+3s)
"22"	1+2s	(1-s_1_)(1+3s)	(1-s_1_)(1+3s)	0

* s is positive selection coefficient. Under the DNR selective model, s_1 _= 0, while under the HI selective model, s_1 _= 1.

where w is mean population fitness; r is the recombination rate between two duplicated loci; μ_neo _is the rate of advantageous mutation; under the DNR selective model, s_1 _= 0, while s_1 _= 1 under the HI selective model.

Based on these ODEs, given μ_neo _= 10^-6^, dynamic changes of chromosomal haplotype frequencies were numerically obtained by the Runge-Kutta method [18] given initial conditions x_0 _= 1, and x_1 _= x_2 _= 0; with considering genetic drift (i.e. in an finite population) simulations were also carried out to test the numerical results.

#### Numerical results

**Figure 1:**
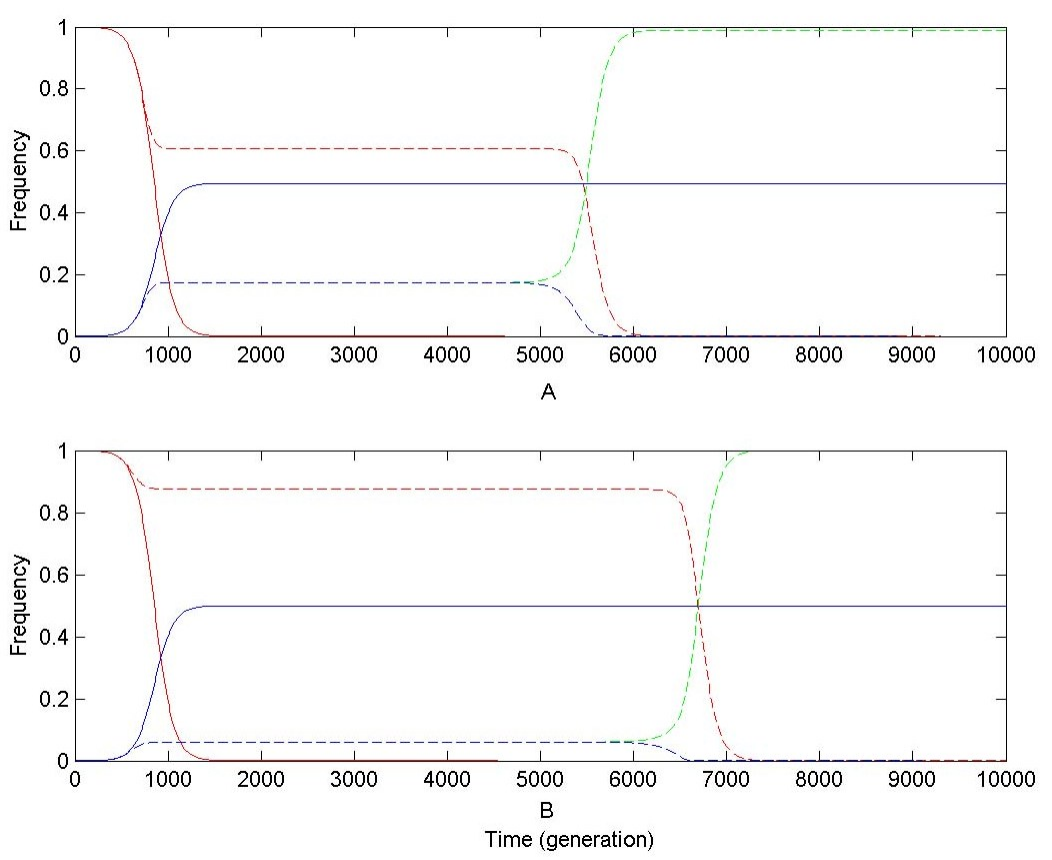
**Dynamic changes of chromosomal haplotype frequencies for gene duplication during neofunctionalization under strong positive selection**. Assume s = 0.01 and μ_neo _= 10^-6^. In subplot **A**, are numerical results under the DNR selective model; in subplot **B**, numerical results under the HI selective model. Solid and dashed curves are numerical results for linked and unlinked gene duplication, respectively. Red, green and blue curves are numerical results for frequencies of chromosomal haplotypes "00", "02" and "20", corresponding to x_0_, x_1 _and x_2_, respectively. In subplots A and B, for linked gene duplication, curves of x_1 _and x_2 _are completely coincident.

In an infinite population dynamic changes of chromosome haplotypes under strong positive selection (s = 0.01) are shown in Figure 1. For linked gene duplication, the frequency of original chromosomal haplotype, x_0_, decreases nearly exponentially down to 0; x_1 _and x_2 _increase continually up to ~0.5. However, for unlinked gene duplication, the behaviors of chromosomal haplotype frequencies are more interesting. Initially, x_0 _decreases to an equilibrium and then is kept at a high level while x_1 _and x_2 _increase also to equilibrium. This equilibrium is kept for a period of time, and then it crashes suddenly, in which x_0 _drops down to very low (close to 0) suddenly, and so does one of x_1 _and x_2 _while another increases up to ~1 (see Figure 1). At neofunctionalization, x_1 _or x_2 _are equal to 1, so these numerical results suggest that in finite and large populations T_neo _for unlinked duplicate genes might be shorter than that for linked. Under recombination high x_0 _in the population was named originalization [15], which descirbes the main difference between evolutionary trajectories of unlinked and linked gene duplications (see Figure 1; also see Ref. [15] and [16]). Therefore, these observations suggest that by originalization, under strong positive selection recombination contribute to shortened T_neo _for unlinked gene duplication.

#### Simulation results

**Figure 2:**
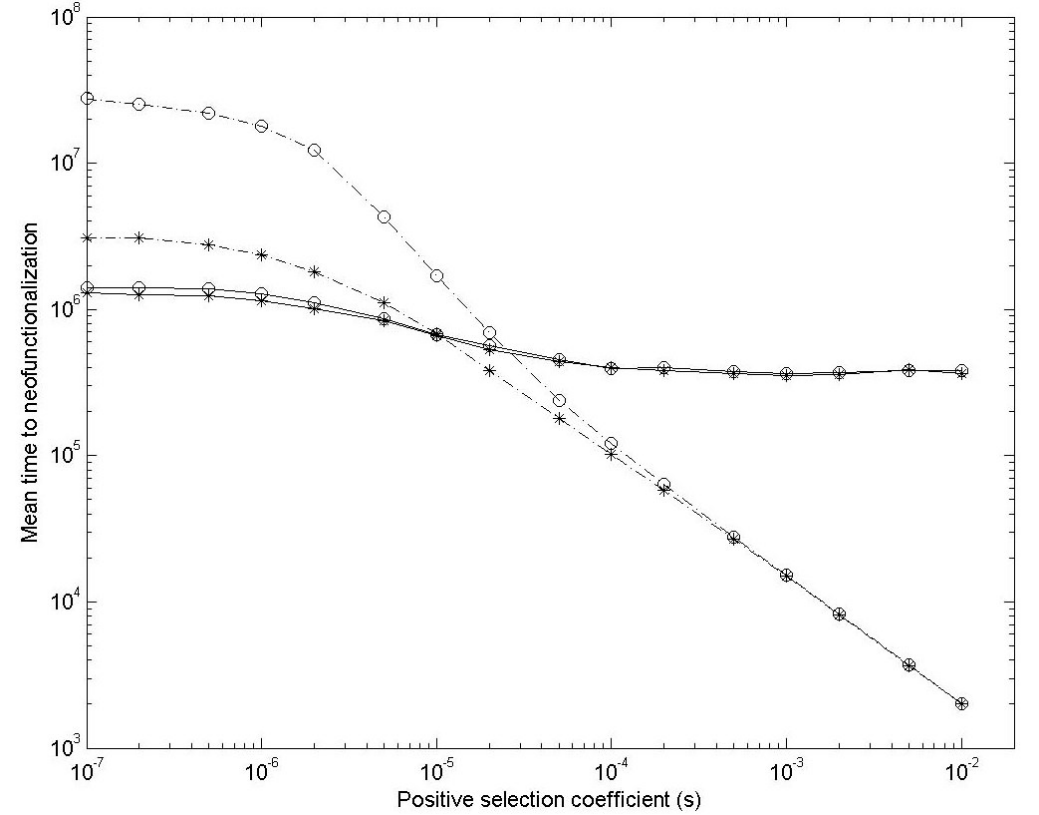
**Simulation results for mean time to neofunctionalization of gene duplication with positive selection coefficient**. Assume N = 200000 and μ_neo _= 10^-6^. Star and circle spots are simulation results under the DNR and HI selective models, respectively. Solid and dash-dot lines are simulation results for linked and unlinked gene duplication, respectively. Simulation repeats 3000 times.

To examine this prediction of shortened T_neo _for unlinked duplicate genes in large populations, simulation results in a larger population (N μ_neo _= 0.2) are shown in Figure 2. Of course, similar results are obtained in other larger populations (N μ_neo _> 0.2) (not shown). However, even when N μ_neo _= 0.2, the results sufficiently indicate that T_neo _for unlinked duplicate genes is shortened when positive selection is strong (see Figure 2).

If s is small enough (or close to 0), the evolutionary behavior of an advantageous mutation is similar to that of a nearly neutral mutation [19]. Therefore, in simulation, when s is small (for example, s = 10^-7 ^in Figure 2) and population size is not small (roughly N μ_neo _> 0.1), T_neo _for unlinked gene duplication is larger than that for linked under the either DNR or HI selective model; and T_neo _for unlinked gene duplication becomes greatly prolonged under the HI selective model (see Figure 2). These observations are very consistent with those of degenerative mutations in previous studies [15,16,20-23]. When s is large (for example, s = 0.01), T_neo _for unlinked gene duplication is much shortened and smaller than that for linked (see Figure 2), which is in agreement with above numerical results.

In our previous studies [15,16], we observed that under recombination T_non _can be prolonged in a larger population (roughly N μ_non _> 0.1); and x_0 _is kept higher in the population. So prolonged T_non_, shortened T_neo _and high x_0 _might jointly result in larger P_neo _for unlinked gene duplication. In order to validate this prediction, direct observations of P_neo _are also carried out.

### Probability of neofunctionalization for gene duplication

#### Model

Now consider a model involving neofunctionalziation and nonfunctionalization. In the gene pool, there are nine possible chromosomal haplotyes in the population, "00", "01", "02", "10", "11", "12", "20", "21", "22", whose frequencies are denoted as y_0_, y_1_, y_2_, y_3_, y_4_, y_5_, y_6_, y_7_, y_8_, respectively. Fitnesses of individuals with various genotypes are shown in Table 2. Under these conditions, in an infinite population another group of ODEs, just like Equation 3, have been obtained. Their expressions are too lengthy, so they are provided in Appendix. Numerical and simulation methods are the same as those in the above section. Numerical and simulation results were also obtained with the rate of degenerative mutation (μ_non_) = 10^-4 ^and that of advantageous (μ_neo_) = 10^-6^. Initially let y_0 _= 1, and y_1 _= y_2 _= y_3 _= y_4 _= y_5 _= y_6 _= y_7 _= y_8 _= 0.

**Table 2:** Fitnesses of individual genotypes for resolution (neofunctionalizaion and nonfunctionalization) of gene duplication*

chromosomal haplotypes	"00"	"01"	"02"	"10"	"11"	"12"	"20"	"21"	"22"
"00"	1	1	1+s	1	1	1+s	1+s	1+s	1+2s
"01"	1	1	1+s	1	1-s_1_	(1-s_1_)(1+s)	1+s	(1-s_1)_(1+s)	(1-s_1)_(1+2s)
"02"	1+s	1+s	1+2s	1+s	(1-s_1_)(1+s)	(1-s_1_)(1+2s)	1+2s	(1-s_1_)(1+2s)	(1-s_1_)(1+3s)
"10"	1	1	1+s	1	1-s_1_	(1-s_1_)(1+s)	1+s	(1-s_1_)(1+s)	(1-s_1_)(1+2s)
"11"	1	1-s_1_	(1-s_1_)(1+s)	1-s_1_	0	0	(1-s_1_)(1+s)	0	0
"12"	1+s	(1-s_1_)(1+s)	(1-s_1_)(1+2s)	(1-s_1_)(1+s)	0	0	(1-s_1_)(1+2s)	0	0
"20"	1+s	1+s	1+2s	1+s	(1-s_1_)(1+s)	(1-s_1_)(1+2s)	1+2s	(1-s_1_)(1+2s)	(1-s_1_)(1+3s)
"21"	1+s	(1-s_1_)(1+s)	(1-s_1_)(1+2s)	(1-s_1_)(1+s)	0	0	(1-s_1_)(1+2s)	0	0
"22"	1+2s	(1-s_1_)(1+2s)	(1-s_1_)(1+3s)	(1-s_1_)(1+2s)	0	0	(1-s_1_)(1+3s)	0	0

* s is positive selection coefficient. Under the DNR selective model, s_1 _= 0, while under the HI selective model, s_1 _= 1.

#### Numerical results

**Figure 3:**
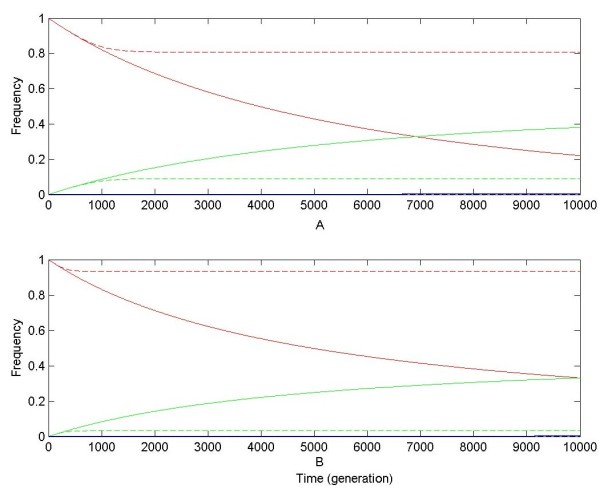
**Dynamic changes of chromosomal haplotype frequencies for gene duplication during resolution (neofunctionalization and nonfunctionalization) under slight positive selection**. Assume μ_neo _= 10^-6^, μ_non _= 10^-4^, and s = 10^-6^. In subplot **A**, numerical results are obtained under the DNR selective model; in subplot **B**, numerical results under the HI selective model. Solid and dashed curves are numerical results for linked and unlinked gene duplication, respectively. Red, gree and blue curves are numerical results for frequencies of chromosomal haplotypes "00", "01" (or "10") and "02" (or "20"), corresponding to y_0_, y_1 _and y_2_, respectively. In subplots A and B, for linked gene duplication, curves of y_2 _are nearly coincident with x-axis.

**Figure 4:**
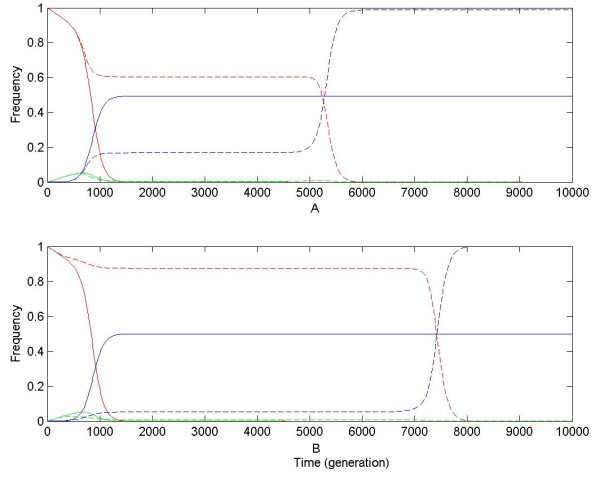
**Dynamic changes of chromosomal haplotype frequencies for gene duplication during resolution (neofunctionalization and nonfunctionalization) under strong positive selection**. Assume μ_neo _= 10^-6^, μ_non _= 10^-4^, and s = 0.01. In subplot **A**, numerical results are obtained under the DNR selective model; in subplot **B**, numerical results under the HI selective model. Solid and dashed curves are numerical results for linked and unlinked gene duplication, respectively. Red, green and blue curves are numerical results for frequencies of chromosomal haplotypes "00", "01" (or "10") and "02" (or "20"), corresponding to y_0_, y_1 _(or y_4_) and y_2 _(or y_6_), respectively. In subplots A and B, for linked gene duplication, curves of y_1 _are nearly coincident with x-axis.

Numerical results are shown in Figure 3 and 4. P_neo _can be approximately expressed as y_2_+y_5_+y_8 _or y_6_+y_7_+y_8_, and the probability of nonfunctionalization as y_1_+y_4_+y_7 _or y_3_+y_4_+y_5_. Because under the DNR and HI selective model described above, y_4_, y_5_, y_7 _and y_8 _are quite small and close to 0, P_neo _is approximately equal to y_2 _or y_6_, and the probability of nonfunctionalization is approximately equal to y_1 _or y_3_. So only dynamic changes of y_2 _and y_1 _are shown in numerical results as the proxies for the probabilities of neofunctionalization and nonfunctionalization, respectively, and y_0 _is treated as a proxy of non-resolution (or originalization) [15].

When positive selection is slight (s = 10^-6^), for unlinked gene duplication, an equilibrium is quickly reached for y_0_, y_1_, and low-level y_2_, while for linked duplication, y_0 _continually decrease with increasing y_1 _and very low (close to 0) y_2 _(see Figure 3). These indicate that under weak positive selection high frequency of original allele and low frequency of advantageous alleles are both buffered on unlinked duplicate loci in the population.

When positive selection is strong (s = 0.01), for linked duplication, y_0 _decreases exponentially down to be very low (close to 0); and y_2 _increase continually up to ~0.5. However, for unlinked gene duplication, y_0 _is only kept high for a period of time and then crashes while y_2 _increases suddenly up to be very high (~1) (see Figure 4), which is very similar to observations in Figure 1. These results, combined with results in the above section and in our previous studies, including high y_0 _and sudden increase of advantageous allele frequency at one of duplicated loci in the population (see Figure 4), prolonged T_non _[15,16,20-23] and shortened T_neo _(see Figure 2), jointly suggest an increase of P_neo _for unlinked gene duplication in finite populations.

#### Simulation results

In finite populations, there are several features in simulation results of P_neo_. First, under strong positive selection, when N is small (roughly N μ_non _< 0.1), P_neo _for unlinked gene duplication under both DNR and HI selective models are all close (see Table 3), and similar to Walsh's prediction - μ_neo_/μ_non _[13,14]. However, when N is larger (roughly N μ_non_) > 0.1), both predictions from Equation 1 and 2 are different from our observations under the DNR selective model in simulation (see Table 3).

**Table 3:** Simulation results for probabilities of neofunctionalization of duplicate genes with different population sizes *

N	DNR_LINK	DNR_FREE	HI_LINK	HI_FREE	Eq_1	Eq_2
100	0.0164	0.0158	0.0182	0.018	0.0392	0.0392
200	0.0236	0.0242	0.0302	0.0394	0.0741	0.077
500	0.0684	0.063	0.0734	0.092	0.1667	0.1832
1000	0.1152	0.1018	0.166	0.2502	0.2857	0.3406
2000	0.1552	0.1646	0.3378	0.6118	0.4444	0.5966
5000	0.5396	0.8696	0.7022	0.9962	0.6667	0.9549
10000	0.9596	0.9998	0.9824	1	0.8	0.9999
20000	0.9996	1	0.9998	1	0.8889	1
50000	1	1	1	1	0.9524	1
100000	1	1	1	1	0.9756	1

* Other genetic parameters are μ_neo _= 10^-6^, μ_non _= 10^-4^, and s = 0.01. Simulation repeats 5000 times. DNR_LINK and DNR_FREE are simulation results on linked and unlinked duplicated loci under the DNR selective model, respectively; HI_LINK and HI_FREE are simulation results on linked and unlinked duplicated loci under the HI selective model, respectively; Eq_1 and Eq_2 are predictions from Equation 1 and 2 of Walsh (1995), respectively.

Second, in a given larger population (N μ_non _= 0.5), simulation results of P_neo _with positive selection coefficient (s) are shown in Table 4. If s is small (roughly Ns ≤ 0.1), P_neo _for unlinked gene duplication under the DNR selective model are also close to Walsh's prediction - μ_neo_/μ_non _[14]. If s becomes larger (roughly Ns > 0.5), P_neo _becomes different from expectations from Equation 1 and 2; and P_neo _for unlinked gene duplication is larger than that for linked under both the DNR and HI selective models (see Figure 4). Therefore, these observations indicate that Equation 1 and 2 don't provide good approximations of P_neo _for unlinked gene duplication under stronger positive selection; and free recombination (r = 0.5) enlarges P_neo_, which is quite consistent with observations of P_neo _in Table 3, in addition to numerical expectations and suggestions in our previous studies [15].

**Table 4:** Simulation results for probabilities of neofunctionalization of duplicate genes with different positive selection coefficients *

s	DNR_LINK	DNR_FREE	HI_LINK	HI_FREE	Eq_1	Eq_2
10^-6^	0.0036	0.0036	0.004	0.0052	0.01	0.0004
10^-5^	0.004	0.0044	0.0054	0.0052	0.0109	0.004
2×10^-5^	0.003	0.0046	0.0048	0.0074	0.012	0.008
5×10^-5^	0.0054	0.0062	0.0066	0.0092	0.0157	0.0198
10^-4^	0.0104	0.008	0.0098	0.0186	0.0226	0.039
2×10^-4^	0.0156	0.0184	0.0206	0.037	0.0392	0.0762
5×10^-4^	0.0444	0.067	0.0566	0.1174	0.0909	0.1774
10^-3^	0.0778	0.1602	0.1338	0.6564	0.16667	0.3177
2×10^-3^	0.1604	0.339	0.244	0.9198	0.2857	0.5212
5×10^-3^	0.3502	0.6584	0.486	0.9872	0.5	0.8161
0.01	0.5412	0.8628	0.6986	0.9958	0.6667	0.9549
0.02	0.752	0.976	0.8848	0.9982	0.8	0.9963
0.05	0.8356	0.9988	0.989	1	0.9091	1
0.1	0.926	0.9998	0.9986	1	0.9524	1

* Other genetic parameters are μ_neo _= 10^-6^, μ_non _= 10^-4^, and N = 5000. Simulation repeats 5000 times. DNR_LINK and DNR_FREE are simulation results on linked and unlinked duplicated loci under the DNR selective model, respectively; HI_LINK and HI_FREE are simulation results on linked and unlinked duplicated loci under the HI selective model, respectively; Eq_1 and Eq_2 are predictions from Equation 1 and 2 of Walsh (1995), respectively.

**Figure 5:**
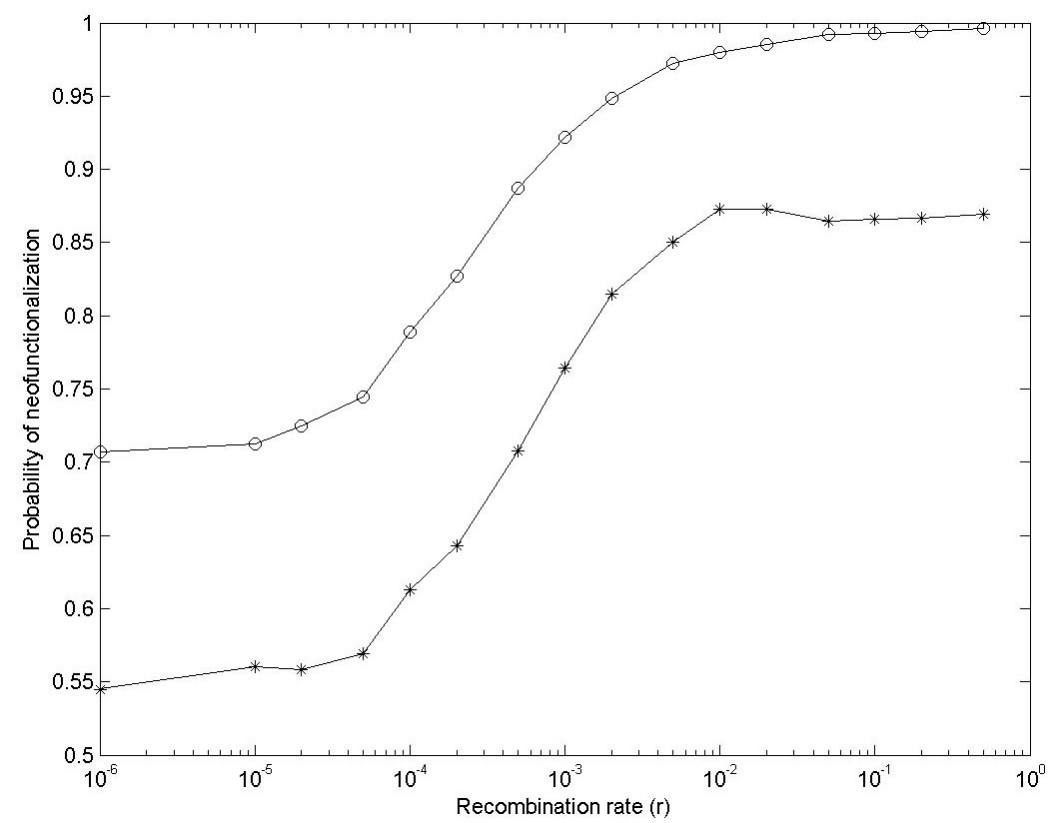
**Simulation results for the probability of neofunctionalization for gene duplication with recombination rate**. Assume N = 5000, μ_neo _= 10^-6 ^and μ_non _= 10^-4^. Star and Circle spots are simulation results under the DNR and HI selective models, respectively.

Third, these observations of P_neo _were obtained under two extreme conditions: linked (r = 0) and unlinked (r = 0.5). However, in most real cases 0 < r < 0.5, so P_neo _with these conditions are also simulated, and results are shown in Figure 5. Simulation results clearly show that as r is larger, P_neo _becomes larger under both DNR and HI selective models. This reinforces our conclusion that recombination enlarges P_neo _under strong selection.

## Discussion and Conclusions

One might argue that these parameters used in above analyses are not realistic enough, for example μ_neo _= 10^-6^, or μ_non _= 10^-4 ^and μ_neo _= 10^-6^. They also can be changed into other more realistic values, for example μ_non _= 10^-6^, and μ_neo _= 10^-9 ^[13,14,23], but these changes do not influence conclusions obtained above except for much prolonged time for calculations.

The sudden crash of the balance of chromosomal haplotype frequencies for unlinked gene duplication in numerical results shown in Figure 1 and 4 might be criticized to result from numerical tolerance. But P_neo _and dynamic changes of genotypes observed directly in simulation are quite consistent with predictions from numerical results. In our previous studies, it has been observed that high x_0 _at the equilibrium can be broken by genetic drift in finite populations [15,16]. In this study this balance can also be broken by strong positive selection.

According to our theoretical results presented in this study and previous studies, several views on the evolution of gene duplication should be revised and reconsidered.

### T_non _might be usually much longer than a few million generations in natural populations

It was commonly considered that for gene duplication, mean time to nonfunctionalization is a few million generations or less (assume degenerative mutation rate is ~10^-6^) [23]. In light of our results, this view should be revised. Only in small populations (N μ_non _≤ 0.01), can mean time to nonfunctionaliztion be simply estimated to be on the order of the reciprocal of degenerative mutation rate for gene duplication - ~ 1/(2 μ_non_) [20,22,23]. However, it increases when population size is larger (roughly N μ_non _> 0.1), especially for unlinked gene duplication [15,16,20-23]. For unlinked haploinsufficient gene duplication, T_non _is prolonged dramatically even in a modest population (0.1 < N μ_non _≤ 1) [15,16]. The underlying mechanism is that under recombination the frequency of original (or wild-type) allele is kept high at both duplicated loci, which is a mathematical process and was named originalization [15,16]. High frequency of original allele (x_0_) in the population retards nonfunctionalization apparently, because at nonfunctionalization x_0 _must be 0. In nature populations, population sizes are usually not small (i.e. N_e _from bacteria is about 10^8^~10^9^, N_e _from yeasts is 10^7^~10^8^, and N_e _from mammals is about 10^4^~10^5^) [24], so T_non _is usually larger than expected in previous studies (~10^6 ^generations).

### Homogenization results from not only gene conversion, but also originalization

Homogenization is often argued to originate from gene conversion. However, in this study, it is observed that under recombination originalization can also result in homogenization. This result is obtained from the principles of traditional population genetics, under a theoretical framework completely different from gene conversion. In our previous studies on originalization, the effect of gene conversion was neglected. Moreover, in originalization, the wild-type allele is buffered with high frequency on both duplicated loci, which retards the divergence of duplicate genes, while in gene conversion, it is not certain that the wild-type allele is converted on duplicated loci. And during gene conversion, d_n _(the rate of non-synonymous nucleotide substitution) and d_s _(the rate of synonymous nucleotide substitution) of duplicate genes are both small. However, in originalization, under purifying selection, d_n _of duplicate genes are small while d_s _are large. This prediction might be applicable to distinguish the effect of originalization from that of gene conversion on genomic evolution.

### P_neo _cannot be expected to be small in natural populations although the rate of advantageous mutation is much small compared with that of degenerative mutation

The rate of degenerative mutation is usually much larger than that of advantageous mutation. So under neutrality, the probability of fixation of advantageous mutations at a locus is much smaller than that of degenerative mutations. This prediction is still hold on for gene duplication under weak selection [13,14]. As shown in Equation 1 from Walsh (1995) [13] and our simulation results (Table 3), for slightly positive selection (Ns < 0.5), P_neo _is equal to ~μ_neo_/μ_non, _regardless of recombination. However, under strong positive selection, in larger populations (N μ_non _> 0.1) P_neo _becomes larger under recombination than that under linkage (see Table 3; and Ref. [13]). The underlying mechanism is that recombination provokes the loss of degenerative mutations and the maintenance of wild-type allele at both duplicated loci in the population. The high frequency of wild-type allele facilitates the arising and accumulating of advantageous mutation, so P_neo _is enlarged. In this way, the power of positive selection is amplified under recombination.

When the evolution of gene duplication is considered in relation to population subdivision (even speciation), the conclusion of P_neo _enlarged under recombination can be reinforced. When advantageous mutations are slightly selective, each of them is buffered in the population at a low frequency for a prolonged period under recombination by originalization. If environments under which subpopulations live are changing and different, they might provide different strong positive selections, under which advantageous alleles might quickly be fixed at the duplicated loci in subpopulations because of shortened T_neo_. Therefore, P_neo _of duplicate genes in nature populations might be larger than expected before.

At the genic level speciation is a differential process accompanied by differential adaptations [25]. It has often been argued that genomic rearrangement resulting from random loss of duplicate genes might cause passive reproductive isolation and then speciation [3,20,26]. Here our results further suggest that via originalization different kinds of neofunctionalizations for duplicate genes among subdivided populations might also contribute to speciation.

## Methods

Methods of simulation and numerical analyses have been described in detail in our previous studies [15,16].

## Authors' contributions

CX conceived of the study, carried out the most works, and drafted the manuscript. YXF participated in the design of the study. RH and SQL performed some simulation works. All authors read and approved the final manuscript.

## Authors' information

Cheng Xue, GuangDong Institute for Monitoring Laboratory Animals, and Key Laboratory of Laboratory Animals in GuangDong, 105 Road Xingang West, Guangzhou, 510260, China. E-mail: lflf27@yahoo.com.cn

Ren Huang, GuangDong Institute for Monitoring Laboratory Animals, and Key Laboratory of Laboratory Animals in GuangDong, 105 Road Xingang West, Guangzhou, 510260, China. E-mail: labking@sohu.com

Shu-Qun Liu, Laboratory for Conservation and Utilization of Bio-resources, Yunnan University,

Yunnan, China. E-mail: shuqunliu@gmail.com

Yun-Xin Fu, Laboratory for Conservation and Utilization of Bio-resources, Yunnan University, Yunnan, China, and Human Genetics Center, School of Public Health, University of Texas at Houston, Houston, Texas USA. E-mail: Yunxin.fu@uth.tmc.edu

## Appendix

Consider a pair of duplicated loci on the same chromosome in a random mating, diploid population without considering genetic drift. Let y_0_, y_1_, y_2_, y_3_, y_4_, y_5_, y_6_, y_7_, y_8 _be the frequencies of chromosomal haplotypes, "00", "01", "02", "10", "11", "12", "20", "21", "22", respectively. The fitness of individual genotypes is shown in Table 2. Under the DNR selective model, s_1 _= 0; Under the HI selective model, s_1 _= 1. Because y_0_+y_1_+y_2_+y_3_+y_4_+y_5_+y_6_+y_7_+y_8 _= 1, only 7 of them are independent. Here we focus on the first 7 frequencies. Therefore, mean population fitness (w) and differential changes of chromosomal haplotype frequencies are given by(A-1)

where r is recombination rate between duplicated loci, s is positive selective coefficient, μ_neo _is advantageous mutation rate and μ_non _is degenerative mutation rate.
